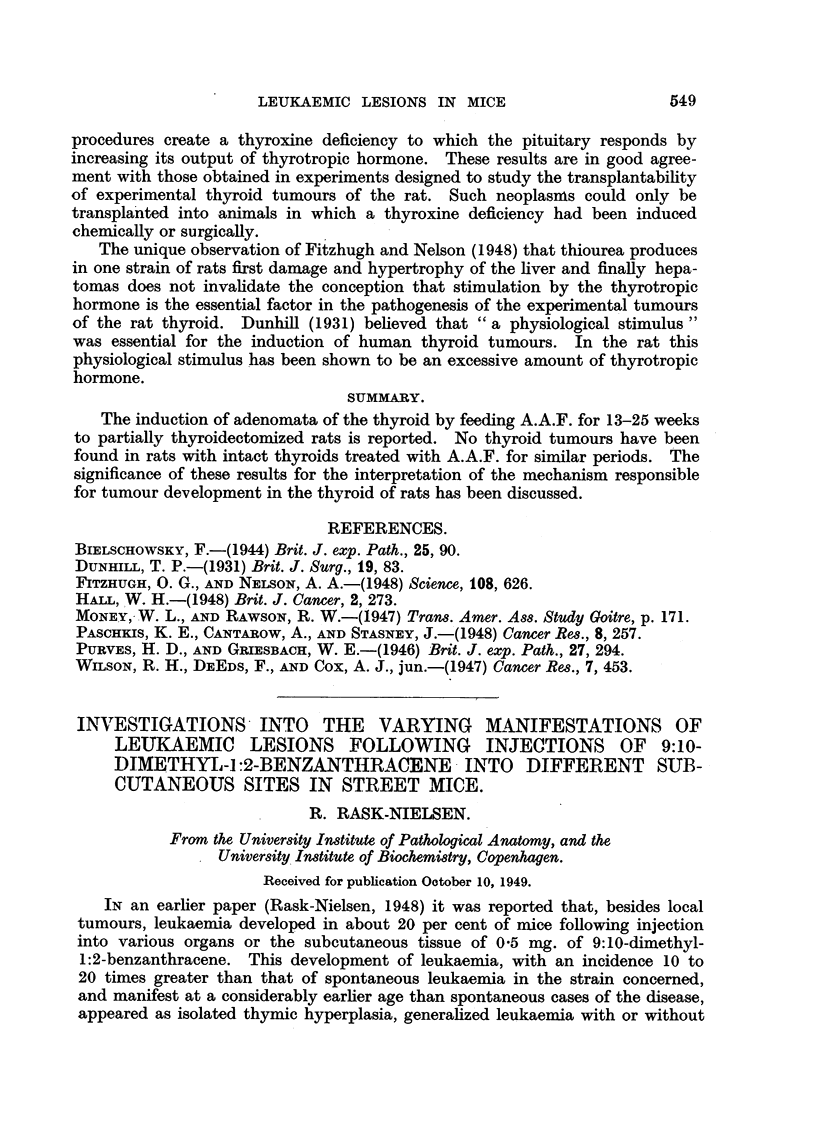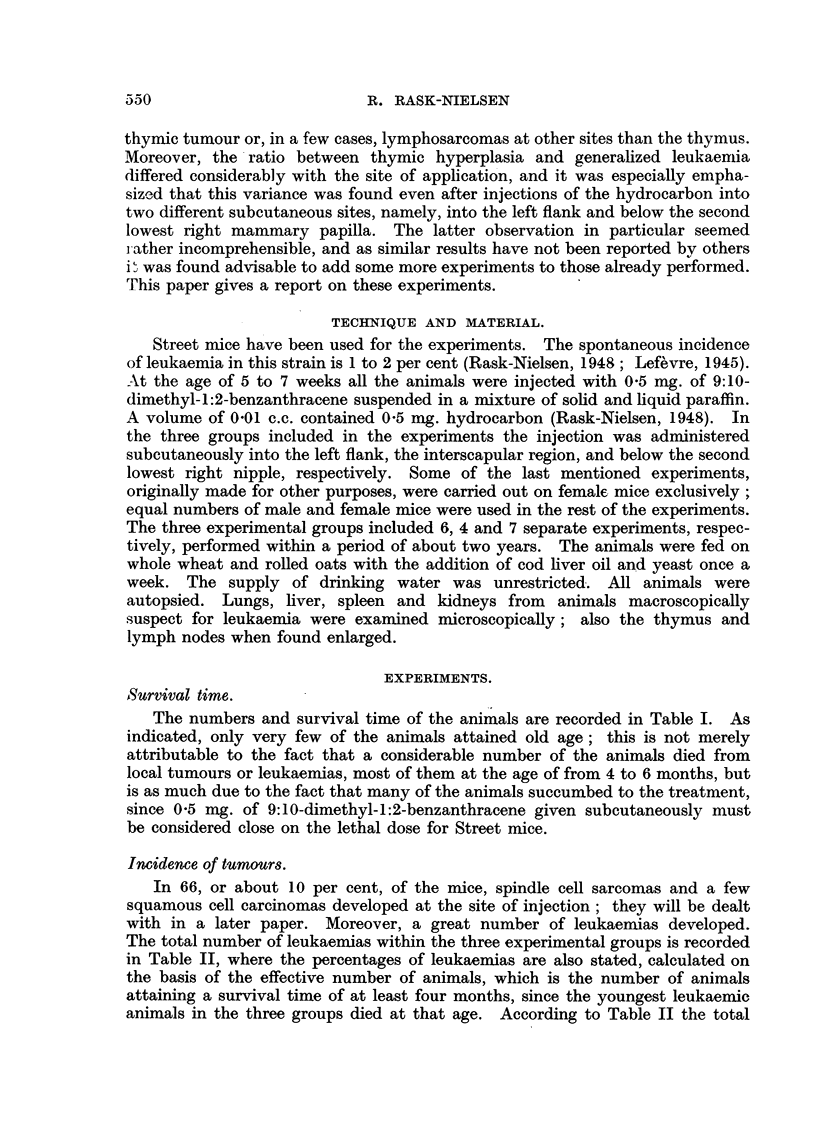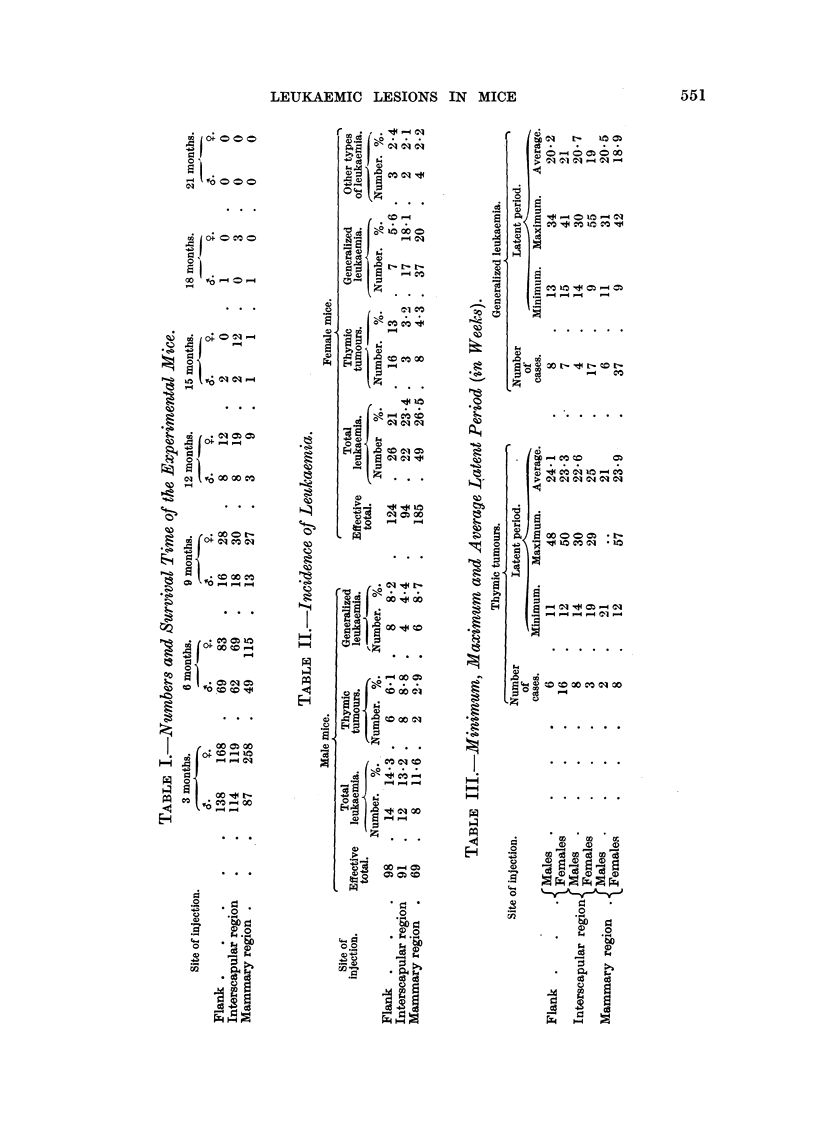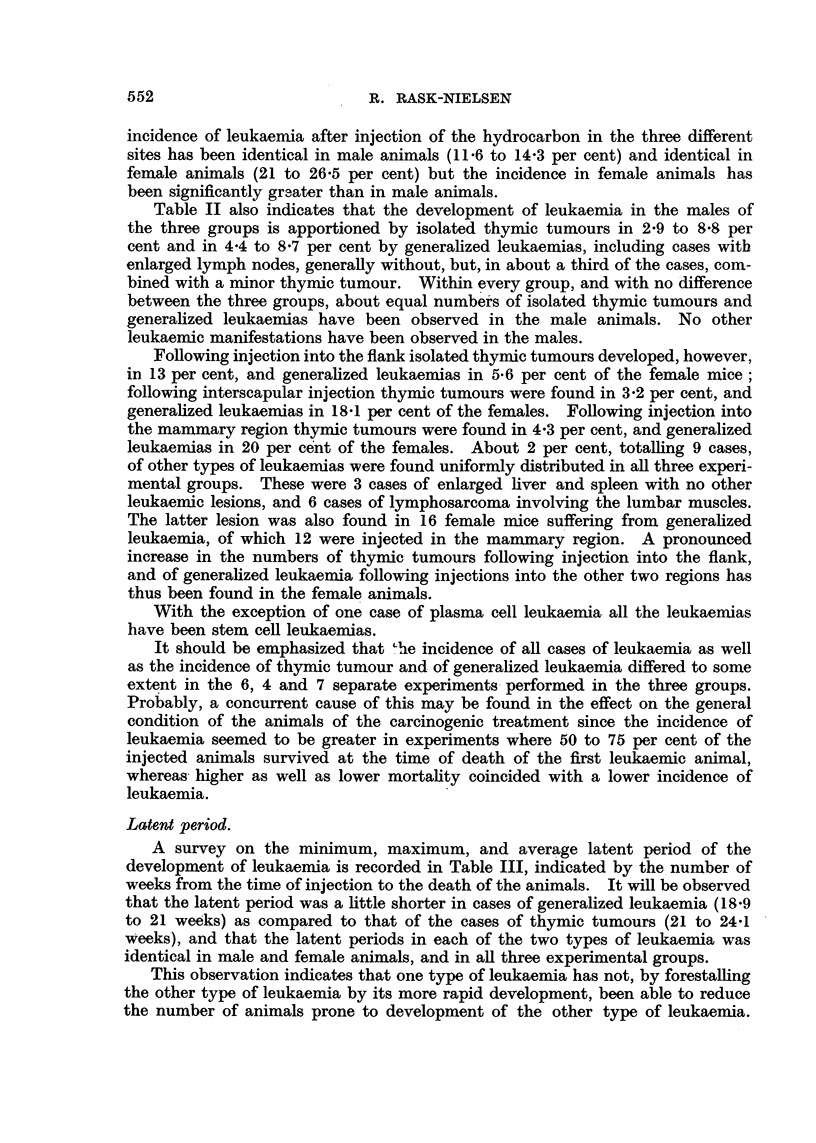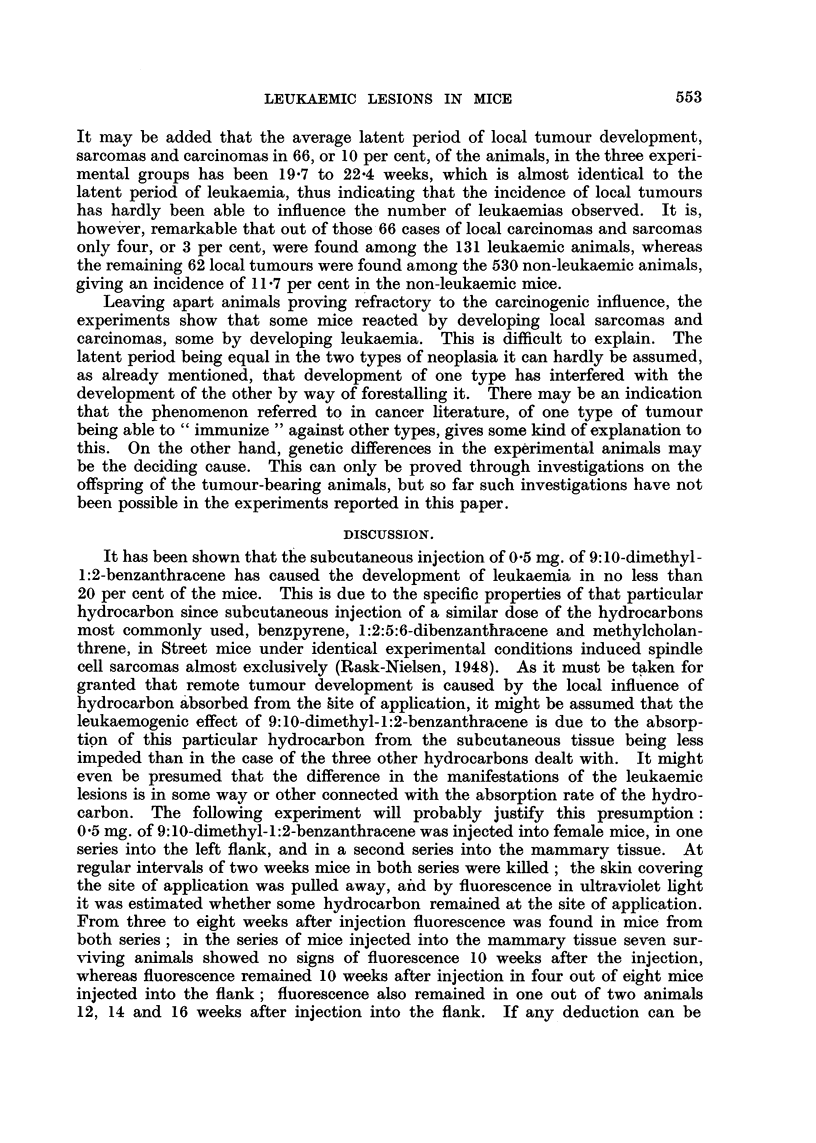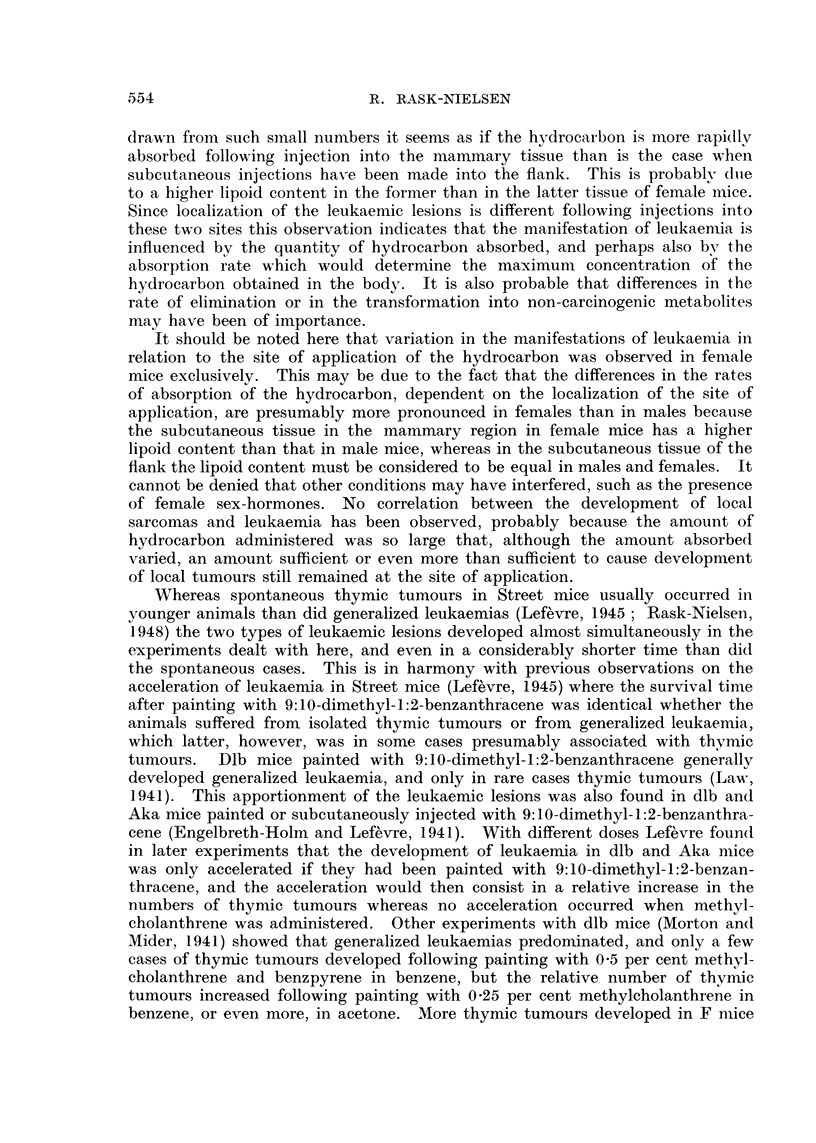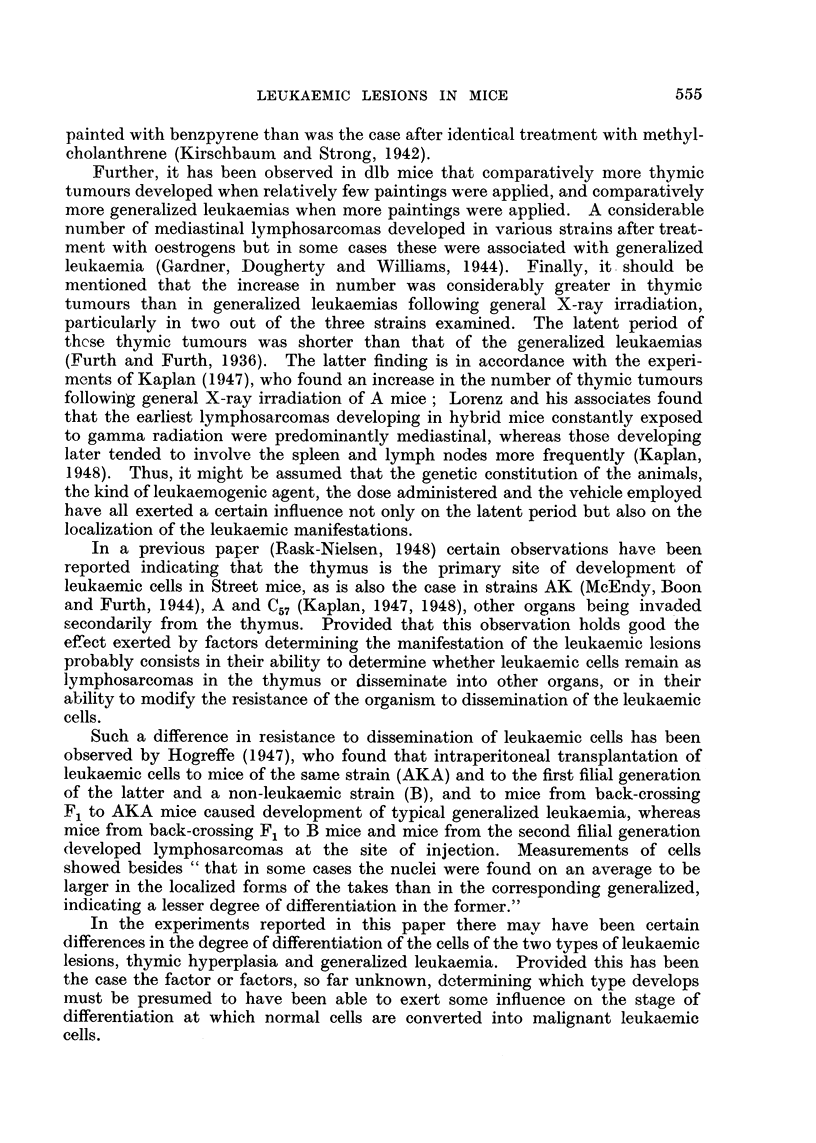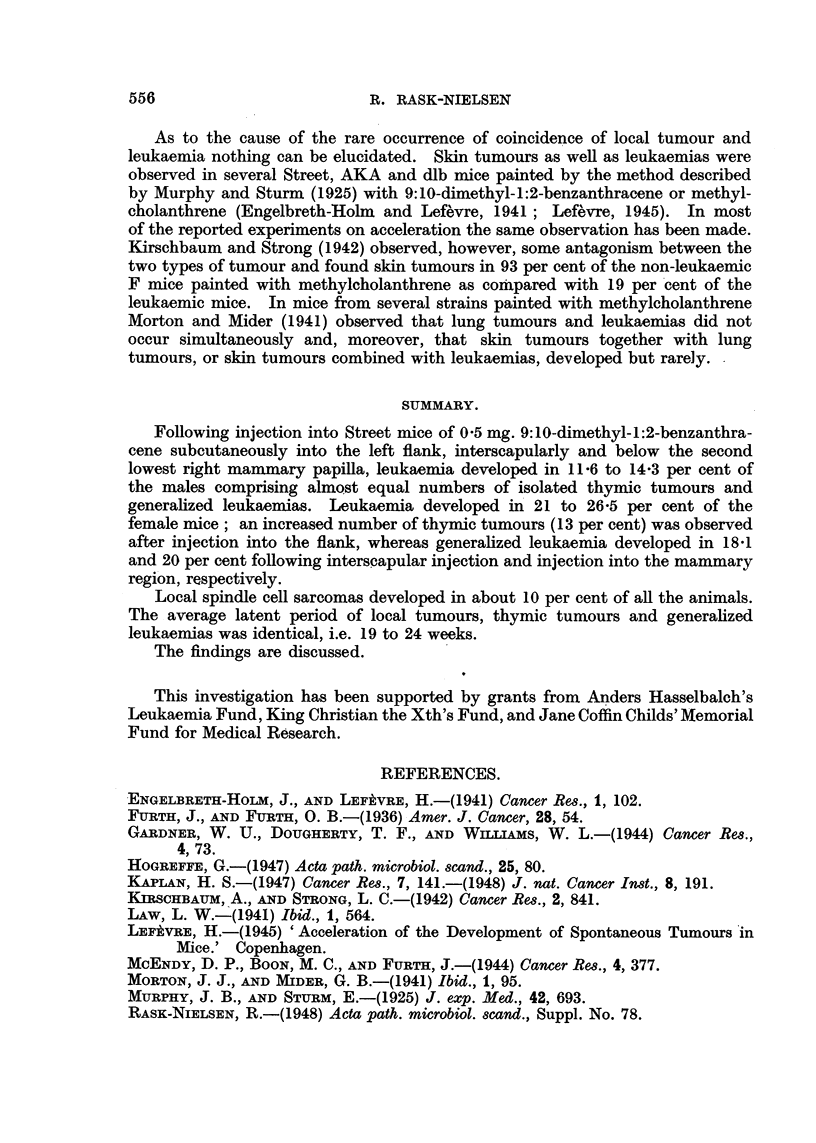# Investigations into the Varying Manifestations of Leukaemic Lesions Following Injections of 9:10-Dimethyl-1:2-Benzanthracene into Different Subcutaneous Sites in Street Mice

**DOI:** 10.1038/bjc.1949.60

**Published:** 1949-12

**Authors:** R. Rask-Nielsen


### INVESTIGATIONS INTO THE VARYING MANIFESTATIONS OF LEUKAEMIC LESIONS FOLLOWING INJECTIONS OF 9:10-DIMETHYL-1:2-BENZANTHRACENE INTO DIFFERENT SUB-CUTANEOUS SITES IN STREET MICE.

R. RASK-NIELSEN.

*From the University Institute of Pathological Anatomy, and the University Institute of Biochemistry, Copenhagen.*

Received for publication October 10, 1949.

In an earlier paper (Rask-Nielsen, 1948) it was reported that, besides local tumours, leukaemia developed in about 20 per cent of mice following injection into various organs or the subcutaneous tissue of 0·5 mg. of 9:10-dimethyl-1:2-benzanthracene. This development of leukaemia, with an incidence 10 to 20 times greater than that of spontaneous leukaemia in the strain concerned, and manifest at a considerably earlier age than spontaneous cases of the disease, appeared as isolated thymic hyperplasia, generalized leukaemia with or without thymic tumour or, in a few cases, lymphosarcomas at other sites than the thymus. Moreover, the ratio between thymic hyperplasia and generalized leukaemia differed considerably with the site of application, and it was especially emphasized that this variance was found even after injections of the hydrocarbon into two different subcutaneous sites, namely, into the left flank and below the second lowest right mammary papilla. The latter observation in particular seemed rather incomprehensible, and as similar results have not been reported by others it was found advisable to add some more experiments to those already performed. This paper gives a report on these experiments.

### TECHNIQUE AND MATERIAL.

Street mice have been used for the experiments. The spontaneous incidence of leukaemia in this strain is 1 to 2 per cent (Rask-Nielsen, 1948 ; Lefèvre, 1945). At the age of 5 to 7 weeks all the animals were injected with 0·5 mg. of 9:10-dimethyl-1:2-benzanthracene suspended in a mixture of solid and liquid paraffin. A volume of 0·01 c.c. contained 0·5 mg. hydrocarbon (Rask-Nielsen, 1948). In the three groups included in the experiments the injection was administered subcutaneously into the left flank, the interscapular region, and below the second lowest right nipple, respectively. Some of the last mentioned experiments, originally made for other purposes, were carried out on female mice exclusively ; equal numbers of male and female mice were used in the rest of the experiments. The three experimental groups included 6, 4 and 7 separate experiments, respectively, performed within a period of about two years. The animals were fed on whole wheat and rolled oats with the addition of cod liver oil and yeast once a week. The supply of drinking water was unrestricted. All animals were autopsied. Lungs, liver, spleen and kidneys from animals macroscopically suspect for leukaemia were examined microscopically ; also the thymus and lymph nodes when found enlarged.

### EXPERIMENTS.

### *Survival time.*

The numbers and survival time of the animals are recorded in Table I. As indicated, only very few of the animals attained old age ; this is not merely attributable to the fact that a considerable number of the animals died from local tumours or leukaemias, most of them at the age of from 4 to 6 months, but is as much due to the fact that many of the animals succumbed to the treatment, since 0·5 mg. of 9:10-dimethyl-1:2-benzanthracene given subcutaneously must be considered close on the lethal dose for Street mice.

### *Incidence of tumours.*

In 66, or about 10 per cent, of the mice, spindle cell sarcomas and a few squamous cell carcinomas developed at the site of injection ; they will be dealt with in a later paper. Moreover, a great number of leukaemias developed. The total number of leukaemias within the three experimental groups is recorded in Table II, where the percentages of leukaemias are also stated, calculated on the basis of the effective number of animals, which is the number of animals attaining a survival time of at least four months, since the youngest leukaemic animals in the three groups died at that age. According to Table II the total incidence of leukaemia after injection of the hydrocarbon in the three different sites has been identical in male animals (11·6 to 14·3 per cent) and identical in female animals (21 to 26·5 per cent) but the incidence in female animals has been significantly greater than in male animals.

Table II also indicates that the development of leukaemia in the males of the three groups is apportioned by isolated thymic tumours in 2·9 to 8·8 per cent and in 4·4 to 8·7 per cent by generalized leukaemias, including cases with enlarged lymph nodes, generally without, but, in about a third of the cases, combined with a minor thymic tumour. Within every group, and with no difference between the three groups, about equal numbers of isolated thymic tumours and generalized leukaemias have been observed in the male animals. No other leukaemic manifestations have been observed in the males.

Following injection into the flank isolated thymic tumours developed, however, in 13 per cent, and generalized leukaemias in 5·6 per cent of the female mice ; following interscapular injection thymic tumours were found in 3·2 per cent, and generalized leukaemias in 18·1 per cent of the females. Following injection into the mammary region thymic tumours were found in 4·3 per cent, and generalized leukaemias in 20 per cent of the females. About 2 per cent, totalling 9 cases, of other types of leukaemias were found uniformly distributed in all three experimental groups. These were 3 cases of enlarged liver and spleen with no other leukaemic lesions, and 6 cases of lymphosarcoma involving the lumbar muscles. The latter lesion was also found in 16 female mice suffering from generalized leukaemia, of which 12 were injected in the mammary region. A pronounced increase in the numbers of thymic tumours following injection into the flank, and of generalized leukaemia following injections into the other two regions has thus been found in the female animals.

With the exception of one case of plasma cell leukaemia all the leukaemias have been stem cell leukaemias.

It should be emphasized that the incidence of all cases of leukaemia as well as the incidence of thymic tumour and of generalized leukaemia differed to some extent in the 6, 4 and 7 separate experiments performed in the three groups. Probably, a concurrent cause of this may be found in the effect on the general condition of the animals of the carcinogenic treatment since the incidence of leukaemia seemed to be greater in experiments where 50 to 75 per cent of the injected animals survived at the time of death of the first leukaemic animal, whereas higher as well as lower mortality coincided with a lower incidence of leukaemia.

*Latent period.*

A survey on the minimum, maximum, and average latent period of the development of leukaemia is recorded in Table III, indicated by the number of weeks from the time of injection to the death of the animals. It will be observed that the latent period was a little shorter in cases of generalized leukaemia (18·9 to 21 weeks) as compared to that of the cases of thymic tumours (21 to 24·1 weeks), and that the latent periods in each of the two types of leukaemia was identical in male and female animals, and in all three experimental groups.

This observation indicates that one type of leukaemia has not, by forestalling the other type of leukaemia by its more rapid development, been able to reduce the number of animals prone to development of the other type of leukaemia.

It may be added that the average latent period of local tumour development, sarcomas and carcinomas in 66, or 10 per cent, of the animals, in the three experimental groups has been 19·7 to 22·4 weeks, which is almost identical to the latent period of leukaemia, thus indicating that the incidence of local tumours has hardly been able to influence the number of leukaemias observed. It is, however, remarkable that out of those 66 cases of local carcinomas and sarcomas only four, or 3 per cent, were found among the 131 leukaemic animals, whereas the remaining 62 local tumours were found among the 530 non-leukaemic animals, giving an incidence of 11·7 per cent in the non-leukaemic mice.

Leaving apart animals proving refractory to the carcinogenic influence, the experiments show that some mice reacted by developing local sarcomas and carcinomas, some by developing leukaemia. This is difficult to explain. The latent period being equal in the two types of neoplasia it can hardly be assumed, as already mentioned, that development of one type has interfered with the development of the other by way of forestalling it. There may be an indication that the phenomenon referred to in cancer literature, of one type of tumour being able to " immunize " against other types, gives some kind of explanation to this. On the other hand, genetic differences in the experimental animals may be the deciding cause. This can only be proved through investigations on the offspring of the tumour-bearing animals, but so far such investigations have not been possible in the experiments reported in this paper.

### DISCUSSION.

It has been shown that the subcutaneous injection of 0·5 mg. of 9:10-dimethyl-1:2-benzanthracene has caused the development of leukaemia in no less than 20 per cent of the mice. This is due to the specific properties of that particular hydrocarbon since subcutaneous injection of a similar dose of the hydrocarbons most commonly used, benzpyrene, 1:2:5:6-dibenzanthracene and methylcholanthrene, in Street mice under identical experimental conditions induced spindle cell sarcomas almost exclusively (Rask-Nielsen, 1948). As it must be taken for granted that remote tumour development is caused by the local influence of hydrocarbon absorbed from the site of application, it might be assumed that the leukaemogenic effect of 9:10-dimethyl-1:2-benzanthracene is due to the absorption of this particular hydrocarbon from the subcutaneous tissue being less impeded than in the case of the three other hydrocarbons dealt with. It might even be presumed that the difference in the manifestations of the leukaemic lesions is in some way or other connected with the absorption rate of the hydrocarbon. The following experiment will probably justify this presumption: 0·5 mg. of 9:10-dimethyl-1:2-benzanthracene was injected into female mice, in one series into the left flank, and in a second series into the mammary tissue. At regular intervals of two weeks mice in both series were killed ; the skin covering the site of application was pulled away, and by fluorescence in ultraviolet light it was estimated whether some hydrocarbon remained at the site of application. From three to eight weeks after injection fluorescence was found in mice from both series ; in the series of mice injected into the mammary tissue seven surviving animals showed no signs of fluorescence 10 weeks after the injection, whereas fluorescence remained 10 weeks after injection in four out of eight mice injected into the flank ; fluorescence also remained in one out of two animals 12, 14 and 16 weeks after injection into the flank. If any deduction can be drawn from such small numbers it seems as if the hydrocarbon is more rapidly absorbed following injection into the mammary tissue than is the case when subcutaneous injections have been made into the flank. This is probably due to a higher lipoid content in the former than in the latter tissue of female mice. Since localization of the leukaemic lesions is different following injections into these two sites this observation indicates that the manifestation of leukaemia is influenced by the quantity of hydrocarbon absorbed, and perhaps also by the absorption rate which would determine the maximum concentration of the hydrocarbon obtained in the body. It is also probable that differences in the rate of elimination or in the transformation into non-carcinogenic metabolites may have been of importance.

It should be noted here that variation in the manifestations of leukaemia in relation to the site of application of the hydrocarbon was observed in female mice exclusively. This may be due to the fact that the differences in the rates of absorption of the hydrocarbon, dependent on the localization of the site of application, are presumably more pronounced in females than in males because the subcutaneous tissue in the mammary region in female mice has a higher lipoid content than that in male mice, whereas in the subcutaneous tissue of the flank the lipoid content must be considered to be equal in males and females. It cannot be denied that other conditions may have interfered, such as the presence of female sex-hormones. No correlation between the development of local sarcomas and leukaemia has been observed, probably because the amount of hydrocarbon administered was so large that, although the amount absorbed varied, an amount sufficient or even more than sufficient to cause development of local tumours still remained at the site of application.

Whereas spontaneous thymic tumours in Street mice usually occurred in younger animals than did generalized leukaemias (Lefèvre, 1945 ; Rask-Nielsen, 1948) the two types of leukaemic lesions developed almost simultaneously in the experiments dealt with here, and even in a considerably shorter time than did the spontaneous cases. This is in harmony with previous observations on the acceleration of leukaemia in Street mice (Lefèvre, 1945) where the survival time after painting with 9:10-dimethyl-1:2-benzanthracene was identical whether the animals suffered from isolated thymic tumours or from generalized leukaemia, which latter, however, was in some cases presumably associated with thymic tumours. Dlb mice painted with 9:10-dimethyl-1:2-benzanthracene generally developed generalized leukaemia, and only in rare cases thymic tumours (Law, 1941). This apportionment of the leukaemic lesions was also found in dlb and Aka mice painted or subcutaneously injected with 9:10-dimethyl-1:2-benzanthracene (Engelbreth-Holm and Lefèvre, 1941). With different doses Lefèvre found in later experiments that the development of leukaemia in dlb and Aka mice was only accelerated if they had been painted with 9:10-dimethyl-1:2-benzanthracene, and the acceleration would then consist in a relative increase in the numbers of thymic tumours whereas no acceleration occurred when methylcholanthrene was administered. Other experiments with dlb mice (Morton and Mider, 1941) showed that generalized leukaemias predominated, and only a few cases of thymic tumours developed following painting with 0·5 per cent methylcholanthrene and benzpyrene in benzene, but the relative number of thymic tumours increased following painting with 0·25 per cent methylcholanthrene in benzene, or even more, in acetone. More thymic tumours developed in F mice painted with benzpyrene than was the case after identical treatment with methylcholanthrene (Kirschbaum and Strong, 1942).

Further, it has been observed in dlb mice that comparatively more thymic tumours developed when relatively few paintings were applied, and comparatively more generalized leukaemias when more paintings were applied. A considerable number of mediastinal lymphosarcomas developed in various strains after treatment with oestrogens but in some cases these were associated with generalized leukaemia (Gardner, Dougherty and Williams, 1944). Finally, it should be mentioned that the increase in number was considerably greater in thymic tumours than in generalized leukaemias following general X-ray irradiation, particularly in two out of the three strains examined. The latent period of those thymic tumours was shorter than that of the generalized leukaemias (Furth and Furth, 1936). The latter finding is in accordance with the experiments of Kaplan (1947), who found an increase in the number of thymic tumours following general X-ray irradiation of A mice ; Lorenz and his associates found that the earliest lymphosarcomas developing in hybrid mice constantly exposed to gamma radiation were predominantly mediastinal, whereas those developing later tended to involve the spleen and lymph nodes more frequently (Kaplan, 1948). Thus, it might be assumed that the genetic constitution of the animals, the kind of leukaemogenic agent, the dose administered and the vehicle employed have all exerted a certain influence not only on the latent period but also on the localization of the leukaemic manifestations.

In a previous paper (Rask-Nielsen, 1948) certain observations have been reported indicating that the thymus is the primary site of development of leukaemic cells in Street mice, as is also the case in strains AK (McEndy, Boon and Furth, 1944), A and $C_{57}$ (Kaplan, 1947, 1948), other organs being invaded secondarily from the thymus. Provided that this observation holds good the effect exerted by factors determining the manifestation of the leukaemic lesions probably consists in their ability to determine whether leukaemic cells remain as lymphosarcomas in the thymus or disseminate into other organs, or in their ability to modify the resistance of the organism to dissemination of the leukaemic cells.

Such a difference in resistance to dissemination of leukaemic cells has been observed by Hogreffe (1947), who found that intraperitoneal transplantation of leukaemic cells to mice of the same strain (AKA) and to the first filial generation of the latter and a non-leukaemic strain (B), and to mice from back-crossing $F_1$ to AKA mice caused development of typical generalized leukaemia, whereas mice from back-crossing $F_1$ to B mice and mice from the second filial generation developed lymphosarcomas at the site of injection. Measurements of cells showed besides " that in some cases the nuclei were found on an average to be larger in the localized forms of the takes than in the corresponding generalized, indicating a lesser degree of differentiation in the former."

In the experiments reported in this paper there may have been certain differences in the degree of differentiation of the cells of the two types of leukaemic lesions, thymic hyperplasia and generalized leukaemia. Provided this has been the case the factor or factors, so far unknown, determining which type develops must be presumed to have been able to exert some influence on the stage of differentiation at which normal cells are converted into malignant leukaemic cells.

As to the cause of the rare occurrence of coincidence of local tumour and leukaemia nothing can be elucidated. Skin tumours as well as leukaemias were observed in several Street, AKA and dlb mice painted by the method described by Murphy and Sturm (1925) with 9:10-dimethyl-1:2-benzanthracene or methylcholanthrene (Engelbreth-Holm and Lefèvre, 1941 ; Lefèvre, 1945). In most of the reported experiments on acceleration the same observation has been made. Kirschbaum and Strong (1942) observed, however, some antagonism between the two types of tumour and found skin tumours in 93 per cent of the non-leukaemic F mice painted with methylcholanthrene as compared with 19 per cent of the leukaemic mice. In mice from several strains painted with methylcholanthrene Morton and Mider (1941) observed that lung tumours and leukaemias did not occur simultaneously and, moreover, that skin tumours together with lung tumours, or skin tumours combined with leukaemias, developed but rarely.

### SUMMARY.

Following injection into Street mice of 0·5 mg. 9:10-dimethyl-1:2-benzanthracene subcutaneously into the left flank, interscapularly and below the second lowest right mammary papilla, leukaemia developed in 11·6 to 14·3 per cent of the males comprising almost equal numbers of isolated thymic tumours and generalized leukaemias. Leukaemia developed in 21 to 26·5 per cent of the female mice ; an increased number of thymic tumours (13 per cent) was observed after injection into the flank, whereas generalized leukaemia developed in 18·1 and 20 per cent following interscapular injection and injection into the mammary region, respectively.

Local spindle cell sarcomas developed in about 10 per cent of all the animals. The average latent period of local tumours, thymic tumours and generalized leukaemias was identical, i.e. 19 to 24 weeks.

The findings are discussed.

This investigation has been supported by grants from Anders Hasselbalch's Leukaemia Fund, King Christian the Xth's Fund, and Jane Coffin Childs' Memorial Fund for Medical Research.

### REFERENCES.

Engelbreth-Holm, J., and Lefèvre, H.—(1941) *Cancer Res.*, **1**, 102.
Furth, J., and Furth, O. B.—(1936) *Amer. J. Cancer*, **28**, 54.
Gardner, W. U., Dougherty, T. F., and Williams, W. L.—(1944) *Cancer Res.*, **4**, 73.
Hogreffe, G.—(1947) *Acta path. microbiol. scand.*, **25**, 80.
Kaplan, H. S.—(1947) *Cancer Res.*, **7**, 141.—(1948) *J. nat. Cancer Inst.*, **8**, 191.
Kirschbaum, A., and Strong, L. C.—(1942) *Cancer Res.*, **2**, 841.
Law, L. W.—(1941) *Ibid.*, **1**, 564.
Lefèvre, H.—(1945) 'Acceleration of the Development of Spontaneous Tumours in Mice.' Copenhagen.
McEndy, D. P., Boon, M. C., and Furth, J.—(1944) *Cancer Res.*, **4**, 377.
Morton, J. J., and Mider, G. B.—(1941) *Ibid.*, **1**, 95.
Murphy, J. B., and Sturm, E.—(1925) *J. exp. Med.*, **42**, 693.
Rask-Nielsen, R.—(1948) *Acta path. microbiol. scand.*, Suppl. No. 78.